# Conformation Study and Design of Novel 6-Hydroxybenzothiazole-2-Carboxamides as Potentially Potent and Selective Monoamine Oxidase B Inhibitors for Neuroprotection

**DOI:** 10.2174/0115680266354743241216065502

**Published:** 2025-01-10

**Authors:** Dong Xie, Penghang Guo, Quantang Zhao, Yu Gao, Jianan Zhang, Jie Zhou

**Affiliations:** 1 Department of Neurosurgery, The 940th Hospital of Joint Logistics Support Force of Chinese People's Liberation Army, Lanzhou, 730000, China;; 2 Department of First Clinical College of Medicine, Gansu University of Traditional Chinese Medicine, Lanzhou, 730000, China

**Keywords:** Neuroprotective activity, 6-hydroxybenzothiazole-2-carboxamide, monoamine oxidase B, QSAR, COMSIA

## Abstract

**Background:**

6-hydroxybenzothiazole-2-carboxamide is a novel, potent, and specific monoamine oxidase B inhibitor that can be used to study the structure of molecules and come up with new ways to protect neurons.

**Objective:**

The objective of this work was to create an effective model using derivatives of 6-hydroxybenzothiazole-2-carboxamide and establish a dependable predictive foundation for the development of neuroprotective monoamine oxidase B inhibitors for the treatment of neurodegenerative diseases.

**Methods:**

The construction and optimization of all compounds were carried out sequentially using ChemDraw software and Sybyl-X software. The optimized compounds were further analyzed using the COMSIA approach and the Sybyl-X software tool for QSAR modeling. A set of novel compounds of 6-hydroxybenzothiazole-2-carboxamide were created and their IC50 values were forecasted using QSAR modeling. Ultimately, the recently developed compounds underwent a screening process using their IC50 values, and molecular docking tests were conducted on the ten most promising compounds with the highest IC50 values.

**Results:**

The 3D-QSAR model exhibited favorable outcomes. The value of q^2^ in the COMSIA model was 0.569. The model demonstrated a superior r^2^ value of 0.915, a lower SEE of 0.109, and a higher F-value of 52.714. The statistical findings and validation of the model were deemed adequate. Furthermore, analyzing the contour plots might assist in identifying the necessary structural specifications.

**Conclusion:**

This work has the potential to provide an insight into the development of active medicines that protect the nervous system against neurodegenerative disorders.

## 1 INTRODUCTION

Neurodegenerative Diseases (NDDs) are a collection of illnesses marked by the gradual deterioration of nerve cells in either the Central Nervous System (CNS) or Peripheral Nervous System (PNS). Parkinson's disease and Alzheimer's disease are the most prevalent examples of such conditions. Parkinson's disease is characterized by the gradual degeneration of neurons, which causes motor system impairments (ataxia). This leads to imbalances, movement problems, and other symptoms, such as resting tremors, muscular stiffness, decreased postural reflexes, and difficulty walking. This condition affects a significant number of individuals globally [1]. As the world's population ages, the occurrence of neurodegenerative illnesses is steadily rising each year on a worldwide scale. It affects around 1% to 3% of those who are 65 years old or older [2, 3]. Presently, there are few effective therapy alternatives available for NDDs [4-6]. Despite the few therapeutic choices available, the success rate remains quite low. To enhance the quality of life and increase the chances of survival for individuals, it is essential to advance the development of novel pharmaceuticals and implement innovative treatment approaches [7, 8].

Neurodegenerative diseases are highly complex and have diverse etiologies, but revealing commonalities in disease mechanisms and pathologies may deepen understanding of the events that precipitate neurodegenerative diseases, such as amyloid deposition, neuroinflammation, and increased oxidative burden [9, 10]. Disorders are associated with an elevation in MAO expression [11]. MAO is an enzyme located on the mitochondrial surface layer and is often referred to as Flavin Adenine Dinucleotide (FAD) [12]. It facilitates the oxidative deamination of diverse naturally occurring amines (such as neuronal, vasoactive, and exogenous amines, along with xenobiotics, including monoamine neurotransmitters and hormones in both brain and peripheral tissues) to modulate their concentrations [13]. It also plays a crucial role in the cellular breakdown of discharged neurotransmitters, which are essential for the proper functioning of synaptic connections [14].

There are two isoforms of MAO (MAO-A and MAO-B). MAO-A is predominantly found in the placenta, intestine, and liver, which is dominated by serotonin and norepinephrine metabolism. MAO-B is predominantly found in the brain, liver, and platelets, which is dominated by the degradation of phenylethylamine, methylhistamine, and tryptamine [15]. The amino acid sequences exhibit homogeneity in 70% of both isoforms [16], and both are involved in the turnover of tyramine and dopamine. Notably, neurotransmitter deficiencies in neuronal tissues increase MAO-B expression, which results in significantly decreasing levels of monoamine transmitters, especially dopamine [17]. MAO facilitates the oxidation of biogenic amines, including dopamine, resulting in the formation of hydrogen peroxide (H2O2), ammonia, and aldehydes, which subsequently generate reactive oxygen species, such as oxidized dopamine and norepinephrine [18]. The main constituents of the Lewy Body (LB) are lipid peroxides and α-synuclein (α-syn) aggregates, and both of these are formed by the production of H2O2, which leads to dyskinesia in PD [7]. Furthermore, the buildup of 3, 4-dihydroxyphenylacetaldehyde (DOPAL) in the brains of Parkinson's disease patients not only degrades synaptic vesicles, but also induces an accumulation of α-synuclein in the terminals of nerves [19]. Consequently, persistently heightened function of the aforementioned enzyme could cause mitochondrial impairment, oxidative stress, and neurodegenerative disorders, including Parkinson's disease and attention deficit disorder, due to biochemical alterations in the concentrations of various neurochemicals within the nervous system that ultimately result in increased cytotoxicity [20].

Hyperphosphorylation of tau proteins causes the development of Neuronal Fibrillary Tangles (NFTs) within neurons, which is a particular change in Parkinson's Disease (PD) [21]. Tau is a protein that binds to microtubules and is mostly found in neurons in the brain [22]. MAO-B activity contributes to the generation of Aβ peptides, which in turn leads to tau phosphorylation [23]. Alpha-synuclein (α-Syn) and tau are significant neuropathogenic proteins that have a crucial function in neurodegenerative disorders [1, 24]. Compounds containing MAO-B have shown encouraging outcomes in the treatment of neurodegenerative disorders, such as Parkinson's disease and Alzheimer's disease [25].

At present, there is a restricted selection of MAO inhibitors that have been approved for commercial usage. These include selegiline [R-(-)-deprenyl] and resazuriline, which work in an irreversible manner [26], as well as safinamide, an MAO inhibitor that functions in a reversible manner [27]. Significantly, selegiline has been linked to orthostatic hypotension and hallucinogenic adverse effects [28]. Safinamide is known to cause birth defects and has also been linked to an increased risk of retinal degeneration. Therefore, it is not recommended to use safinamide during pregnancy or in those with retinal illness [29].

The extreme toxicity and adverse effects of these clinical studies have imposed limitations. In order to tackle this issue more effectively, a range of derivatives of the novel compound 6-hydroxybenzothiazole-2-carboxamide have been discovered. These derivatives involve modifications to the amide substituent, resulting in several powerful compounds with various side chains. These compounds have demonstrated distinct effects in selectively inhibiting MAO-B, making them potential targeted therapeutic agents for neurodegenerative diseases.

Quantitative Structure-activity Relationship (QSAR) is a scientific methodology that involves modeling and predicting the biological activity of a molecule based on quantitative connections between its structural characteristics [30]. Computer-aided drug design has evolved into a mature and promising field of study, focusing on quantitative structural connections [31]. The conventional approach to drug design is marked by inefficiency, lengthy processes, and exorbitant expenses. Consequently, there is a pressing need for novel research and development methodologies to enhance the efficiency and effectiveness of drug design [32]. Computer-aided drug design has gained widespread recognition and use in the field of drug discovery and development due to its notable benefits of increased efficiency, reduced time requirements, and lower costs [33, 34]. The current investigation included the development of a three-dimensional quantitative structure model for novel derivatives of 6-hydroxybenzothiazole-2-carboxamides. This was achieved by the use of Comparative Molecular Similarity Indices Analysis (COMSIA) and molecular docking investigations. This approach has significantly facilitated the comprehension of the correlation between the structure and activity of the chemicals. Based on the findings of QSAR and molecular docking, we may enhance the activity of drugs by making further structural modifications.

## 2 MATERIALS AND METHODS

### 2.1 Experimental

#### 2.1.1 Obtaining and Analyzing Datasets

This research presented the structures and IC50 values of 36 newly discovered compounds of 6-hydroxybenzothiazole-2-carboxamide (Table **1**) [35]. In order to mitigate the asymmetry of the dataset, the IC50 values of all the substances were transformed to -log(IC50) + 6. Afterward, the datasets were randomly split into a training set consisting of 29 compounds and a test set consisting of 7 compounds. The training set was used to develop the 3D-QSAR model, whereas the test set was employed to verify the model.

#### 2.1.2 Structural Optimization

The diagram was drawn using ChemDraw and saved in XX.mol format. The Moe software was used to convert the format from XX.mol to XX.mol2. The target folder was opened (File → open), and the path where the folder was located was copied (no Chinese characters appeared in the path); it was then entered and clicked (CWD → Yes). Afterward, a double-click was done to open the text format that needed to be converted (File → open). XX.mol2 format was selected, and the file was named and saved. Every time a file format needed to be converted, the structure was closed and then the next file was opened for format conversion. After all the compound file formats were converted, the file was opened in mol2 format in Notepad (txt) and the naming was modified (this step was important to make the compound number, structure, and activity data correspond to each other when importing the compound structure into the Sybyl software, so as to avoid any mismatch).

Sybyl software was opened and the default file was set (the path did not contain Chinese characters), and then Options → Set → Default Directory were clicked to select the folder where all the mol2 files were located. File→Import File was clicked to open the molecule to be optimized (double click or select→OK). Parameter setting was performed as follows: after clicking Compute→Minimize→Molecule, max iterations were set to 1000, the option Modify was clicked to open the dialog box, the Gasteiger-Huckel was selected in the Charges drop-down menu, OK was clicked to return to Minimize interface, and then OK was clicked to start the structure optimization. After the optimization was completed, File → Export File was clicked to export the optimized structure. After each structure was optimized, →Delete Everything was clicked.

The IC50 values were log-transformed (μM → -LogIC50+6) to pIC50. All the activity data were organized into Notepad and saved as pIC50.txt. All the optimized compounds in mol2 format were randomly partitioned into the training set (folder training.mdb) and the test set (folder test.mdb). Training.mdb, test.mdb, and pIC50.txt were saved in the same folder.

#### 2.1.3 Compound Superposition

In the 3D-QSAR experiment [36], the Sybyl software was opened and the default file was set. Options→Set→Default Directory were clicked to select the folder where all the data were located and then →OK was clicked. File→Import File was clicked and then Database in the Files of Type drop-down menu was selected, the training.mdb file was selected, and finally, OK was clicked on. The list of training set compounds was opened. In the training form, File→Import was clicked, and a dialog box appeared. The Delimited file in Files of type was selected, the pIC50.csv file was selected, and OK was clicked. A dialog box appeared, and Merge was clicked to import the pIC50 values of the compounds into the list of training set.

**Fig. (1):**
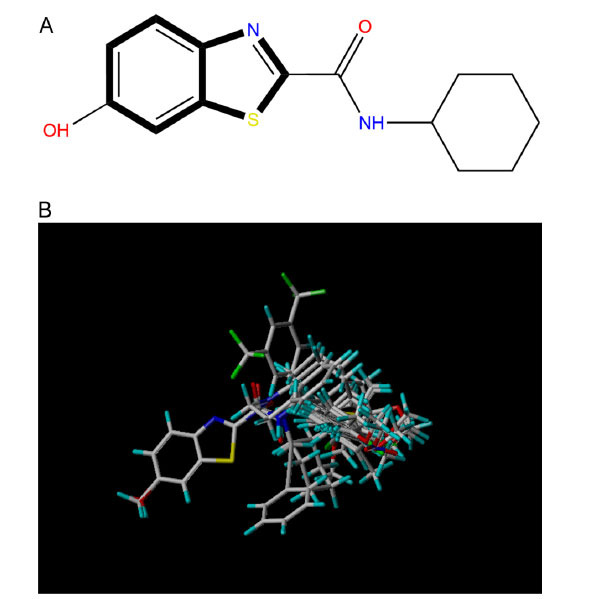
(**A**) Compound superposition using the common skeleton of compound 31. The bold portions represent the universal structure shared by all compounds. (**B**) All compounds were organized based on a shared structure.

The Maximum Common Skeleton Overlay method was adopted by clicking File→Database→Align Database. The template molecules were selected to select the common skeleton and →OK was clicked. Afterward, Apply was clicked to start the automatic overlay (Fig. **1**). When the stacking was finished, a naming window popped up, named training01 (customized), and → OK was clicked. The training form was closed, the training01 file was opened, and finally imported into pIC50 format.

The structural arrangement of a chemical is the key factor that directly impacts the testing of the drug in 3D-QSAR analysis [37]. Compound combinations may be categorized into two distinct forms: one is centered on ligand-based common structures, while the other focuses on receptor-based small molecule targets. The former was used in accordance with the study's needs (Fig. **1**). In this methodology, all compounds were aligned with the prominent structure found in compound 31, which exhibited the highest level of pharmacological activity in this trial.

#### 2.1.4 Study Conducted using COMSIA

A grid with a uniform side length of 0.2A was established to facilitate COMSIA experiments at the locations where the molecules were built. Additionally, a 4A border was used to identify all sections of the stacked molecules. For each grid point, the steric, electrostatic, hydrophobic, and hydrogen bonding fields were calculated. This calculation included the identification of hydrogen bond acceptors and hydrogen bond donors. The default probes provided with COMSIA were used for this computation. A quantitative correlation model was developed using Partial Least Square (PLS) to analyze the relationship between molecular field features and affinity. The molecular field at each grid point was obtained prior to creating the model. The statistical significance of the model was evaluated by the use of leave-one-out and cross-validation approaches. Additionally, the ideal number of key components for the model was determined. A 3D-QSAR model was developed by using a set of key components that were chosen based on the most favorable interaction check outcomes. The model was constructed without including interaction checks and thereafter used to forecast the affinity of the compounds in the test set [26, 27].

#### 2.1.5 Verification of the 3D-QSAR Model

Generally, greater values of q^2^, R^2^, and F, together with lower values of SEE, indicate a good capacity to fit the data. Nevertheless, relying just on these statistical metrics does not completely showcase the model's predictive capacity. Additional validation is necessary to thoroughly assess the model's dependability, resilience, and predictive power [36].

#### 2.1.6 Validation from an External Source

There are two often-used external validation techniques that utilize the following formulae:


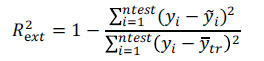


The equation involves the variables ntest, 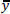
_tr_, and 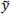. The variable ntest reflects the quantity of compounds in the test set, whereas 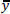
_tr_ represents the mean value of the compound’s activity. The variable ntest denotes the number of compounds in the test set. The variable 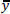
_tr_ represents the average value of compound activity in the training set. The variables 𝑦_𝑖_ and 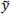 indicate the experimental and expected values of compound activity in the test set, respectively. In the training set, 𝑦_𝑖_ represents the compound activity values. In the test set, 𝑦_𝑖_ represents the experimental values and 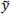
_𝑖_ represents the expected values [38].

Furthermore, the parameter R^2^_m_ may be further validated to assess the appropriateness of the model using the following equation:


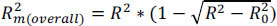


R^2^ in this equation denotes the squared value of the correlation coefficient, which measures the degree of correlation between the anticipated and experimental activity levels for all substances. Conversely, R^2^_0_ is the squared correlation coefficient between predicted and experimental values when the intercept is adjusted to 0.

The R^2^_m_ of the ideal model should be more than 0.5.

## 3 RESULTS AND DISCUSSION

### 3.1 Statistical Findings Derived from the COMSIA Model

The COMSIA approach generates five distinct molecular force fields with various levels of contribution. The proportions of these five force fields are as follows: spatial force (17.2%), electrical force (27.1%), hydrodynamic force (46.2%), hydrogen bond donor (4.5%), and hydrogen bond acceptor (5.0%). By organizing and merging all five of these molecules' fields of force, a total of 10 sets of models were created. Upon careful analysis of these 10 sets of models, it was concluded that the ESHAD model exhibited the highest level of satisfaction, as shown by the findings presented in Table **2** [statistical parameters of the COMSIA model (S: steric, E: electrostatic, H: hydrophobic, D: hydrogen bond donor, A: hydrogen bond acceptor)]. Fig. (**2**) (the force field contribution of the most optimal COMSIA model) illustrates the effect of this model. The most efficient COMSIA model yielded a q^2^ statistic of 0.569, a superior r^2^ value of 0.915, an optimal group score of 3, a reduced SEE of 0.109, and an elevated F value of 52.714.

The q2 value is the cross-validation coefficient, which is used to assess the internal prediction ability of the model. q2 value of 0.569 indicates that the model performs well in internal validation. r2 value is the coefficient of determination, which is used to measure how well the model fits the data in the training set. r2 value of 0.915 shows an extremely high degree of fit. SEE value is the standard error estimate, which reflects the deviation of the model-predicted values from the actual values. The smaller the SEE value, the higher the predictive accuracy of the model. In our model, the SEE value was 0.109, indicating the predictions to be quite accurate. F-value is an F-test statistic used to test the overall significance of the model. The larger the F-value, the more significant the model is. The F-value of our model was 52.714, showing the high significance of the model.

### 3.2 Results of COMSIA Model Validation

The experiment included doing external validation of the COMSIA model. The model that was developed was utilized to predict the behavior of the compounds in the experimental group. This resulted in an R^2^_ext_ value of 0.688, which was calculated using the specified formula. The R^2^_ext_ value beyond 0.5 suggests that the model built is strong and has significant statistical forecasting capability.

The experimental values of the model provided in Fig. (**3**) exhibited a strong linear correlation with the anticipated values.

### 3.3 Contour Maps Generated with COMSIA

**Fig. (4):**
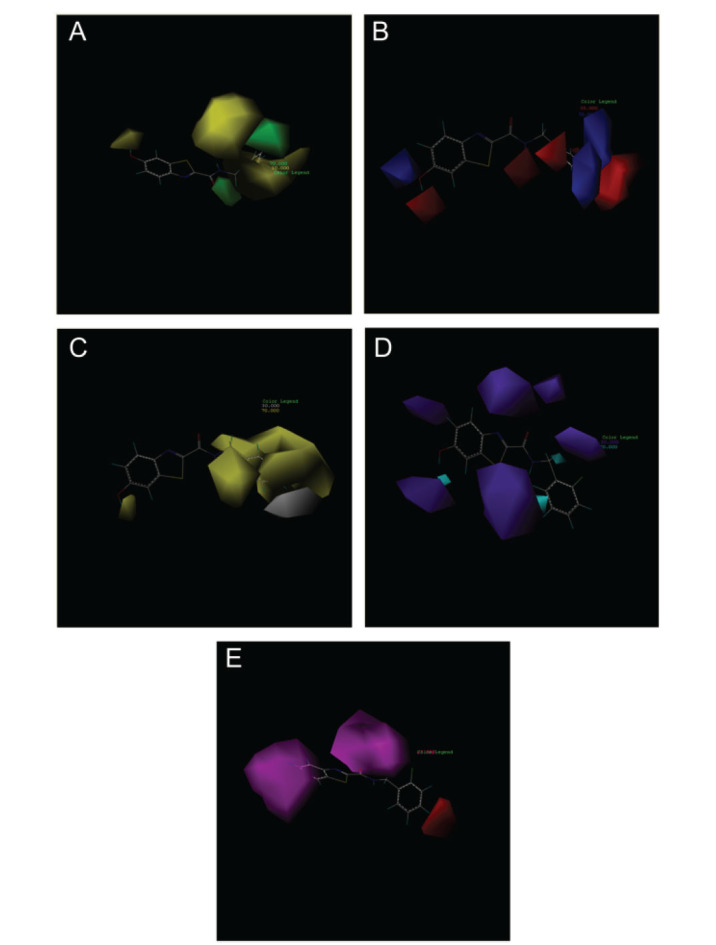
Contour map showing the optimal configuration of compound 31. (**A**) In the steric field, the green color indicates a favorable situation, whereas yellow indicates an unfavorable one. (**B**) In an electrostatic field, the blue color indicates a positive field, whereas the red color indicates a negative field. (**C**) In a hydrophobic environment, the yellow color indicates a favorable condition, whereas white indicates an unfavorable one. (**D**) Purple represents advantageous hydrogen bond donor fields, whereas cyan represents unfavourable hydrogen bond donor fields. (**E**) Favorable (magenta) and unfavorable (red) hydrogen bond acceptor regions.

The contour plots of the COMSIA model provide an insight into the precise correlation between the compound's structure and its pharmacological action (Fig. **4**) [39]. The experiment included creating several contour plots of the molecular steric field, electrostatic field, hydrophobic field, hydrogen bond donor field, and hydrogen bond acceptor field using the StDev*Coeff technique. The relationship between compound structure and pharmacological action was discerned by the observation of color variations in various areas of the contour plots [40].

Compound 31 was chosen as the designated structure for the COMSIA model contour plot, as can be seen in Fig. (**4**). Fig. (**4A**) displays the green contour in the vicinity of the R^1^ position, which signifies a favorable spatial area where the existence of substantial group substituents amplifies the biological activity. Furthermore, the presence of the yellow contour next to the R^1^ location suggests that even minor alterations of functional groups might potentially impact the activity. The blue contour around point R^1^ in Fig. (**4B**) shows the existence of an electrostatic field. Hence, the introduction of cationic substituents at this location may enhance the biological activity. The yellow outline around the R^1^ position in Fig. (**4C**) represents the hydrophobic boundary. This suggests that the presence of hydrophobic groups at this location intensifies the effect. The presence of a white contour implies that the hydrophilic group plays a substantial role in the biological activity. The inclusion of a hydrophilic group at this location amplifies its effectiveness. Compound 31 molecule is situated at the R^1^ position and exhibits more activity in comparison to the other compounds. Both Fig. (**4D** and **4E**) have contours at the R^1^ location, showing the existence of hydrogen bonding. These contours indicate the orientation for the inclusion or removal of hydrogen bond donor or acceptor groups. The contour plots display hydrogen bond acceptors using fuchsia and red contours. Placing a hydrogen bond acceptor group next to the fuchsia contour enhances activity, whereas the red contour indicates regions where a hydrogen bond acceptor group is unnecessary. The contour map representing the hydrogen bond donor is shown by the purple and blue-green contours. Introducing a hydrogen bond donor group close to the purple contour is advantageous for enhancing biological activity, whereas placing a hydrogen bond donor group near the blue-green contour does not provide the intended outcome.

### 3.4 Developed Novel Compounds and Forecasted IC50 Values using the 3D-QSAR Model

The neuroprotective efficacy of the targeted medicine in MAO-B neurodegenerative illness was determined based on the primary parameters impacting it. Structural alteration of the reference molecule 31 was conducted by following the contour lines and combining distinct molecular fields in the contour plot. Upon consolidating the contour findings, we designated the locations to be altered as R^1^ and R^2^. Due to the fact that hydrophobic fields increase the efficacy of the medicine, we used hydrophobic groups for structural modification. Hydrophobic groups include several types of chemical groups, such as alkyl groups (including -CnH2n +1, -CH=CH_2_, C_6_H_5_, *etc*.), halogen atoms (-X), and nitro group (-NO2), among others. Utilizing these modification principles, we have successfully produced over 100 novel chemicals. A total of over 100 compounds were optimized using SYBYL 2.1.1, and their pIC50 values and overall scores were predicted. The top-performing compounds, namely compounds 31.a-31.j, were chosen for further investigations. Table **3** displays the molecular structures of these 10 compounds together with their estimated pIC50 values and overall scores. The data indicated that compound 31.j exhibited the greatest pharmacological activity value, suggesting it to have significant potential for neuroprotective action.

### 3.5 Findings from Molecular Docking

The drug molecules were imported into the Syphilis program, and the molecular mechanics of the small ligand particles were improved utilizing a conjugated cascade technique. The optimization procedure was executed with the Tripos force field, with the energy convergence criteria set at 0.01 kcal/(mol-A) and a maximum iteration limit of 106. Following the optimization of molecular mechanics, the most favorable active conformation was chosen for molecular docking investigations. We obtained a subset of the MAO-B receptor (MAO-B crystal structure, PDB ID: 3PO7) from the RCSB PDB protein database by isolating it from the water molecules and hydrogen atoms present in the crystal structure. This subset will be used for molecular docking in the future. Following that, the first ligand in the MAO-B fragment was separated and the precise position of its binding site was identified, as shown in Fig. (**5**).

The Sybyl-X tool is used for performing variable docking among tiny molecules and receptors. Active pockets are formed by the identification and use of the binding site of the target ligand. The threshold parameter was assigned a value of 0.5, the growth factor was assigned a value of 1, and the docking method was executed using the Sybyl-Dock standard mode. The molecular conformational changes were maintained for a duration of 20 units of time. The analysis of the relationship between the small molecule and the object being studied was conducted using the overall scoring given by the Sybyl-Dock tool. Higher values suggest more potent relationships among drug molecules with protein crystals.

Based on Fig. (**1**) and COMSIA contour plots, it was evident that the primary parameters influencing the activity of 6-hydroxybenzothiazole-2-carboxamide derivatives were hydrophobicity and hydrogen bonding. Consequently, the development of novel chemicals should be derived from this. Nevertheless, the docking test findings revealed that a portion of the 200 recently developed compounds had either low activity levels or were incapable of successfully completing docking with the tiny MAO-B molecules in space, as indicated by their extremely low docking scores. Through careful analysis of these matters, it was discovered that the creation of novel chemicals is conducted at the two-dimensional scale. Hence, when compounds undergo three-dimensional folding, there is a potential for misalignment with tiny MAO-B molecules, resulting in diminished activity levels and docking scores. Compound 31.j was an archetypal illustration. Fig. (**5**) indicates that compound 31.j exhibited the ability to fold in space, resulting in the formation of a ring-shaped structure. This structure could efficiently bind to the specific region of the MAO-B segment. Moreover, compound 31.j demonstrated the ability to form hydrogen bonds with three particular residues of the MAO-B fragment: GLU-34, ARG-42, and LEU-268. Unlike other compounds, several compounds had the ability to create just two, one, or even no bonds. Compound 31.j had the highest docking prediction score (Table **3**), consistent with the findings. Compound 31.j can be regarded as a promising candidate because of its ability to protect the nervous system from damage in neurodegenerative diseases.

## 4 DISCUSSION

The q^2^ value of the COMSIA model in this study was 0.569, indicating the model to have a good internal prediction ability. This value is usually considered an important indicator of the model's predictive reliability, indicating that the model has high stability and predictive accuracy for the training set data. r^2^ value was 0.915, showing that the model fit the training set data very well, further proving the model's predictive ability. The Standard Error of Estimate (SEE) was 0.109, indicating the deviation between the model's predicted value and the experimental value to be small; the F value was 52.714, which showed the model to have high statistical significance.

The different contribution proportions of the spatial (17.2%), electrostatic (27.1%), hydrophobic (46.2%), hydrogen bond donor (4.5%), and hydrogen bond acceptor (5.0%) fields in the model illustrated the different degrees of influence of each molecular field on the activity of the compounds. In particular, the high percentage contribution of the hydrophobic field (46.2%) implied the importance of hydrophobic interaction in determining the activity of these compounds.

Highly active compounds (*e.g*., compound 31.j) obtained by QSAR model prediction provided powerful candidates for new drug discovery, demonstrating the potential of model application in new drug discovery.

### 4.1 Limitations of External Validation

The test set contained only seven compounds, a relatively small sample size that may affect the accuracy and reliability of external validation. Future studies should expand the size of the test set to further validate the generalizability of the model. *Limitations of model applicability*. Structural type limitation: the current model was only optimized for 6-hydroxybenzothiazole-2-carboxamide derivatives of a specific structure and may not be applicable to other structural types of compounds. Therefore, the model needs to be reconstructed and optimized before applying it to new structure types.

Compared to existing MAO-B inhibitors (*e.g*., selegiline, rasagiline), the 6-hydroxybenzothiazole-2-carboxamide derivatives developed in this study may exhibit higher selectivity while maintaining a high inhibitory activity against MAO-B, thus reducing the effect on non-target enzymes [35]. Existing inhibitors may be accompanied by side effects, such as postural hypotension and hallucinations, while the new compounds are expected to reduce the occurrence of these adverse effects through structural optimization. The new compounds are designed with pharmacokinetic optimization in mind and may have better oral absorption, longer half-life, and higher bioavailability.

In this study, 6-hydroxybenzothiazole-2-carboxamide was used as the backbone, and a series of novel derivatives were obtained by structural modification, which were significantly different from the structures of MAO-B inhibitors commonly used in previous studies, and provided new molecular entities for drug development. Biological activity prediction using the 3D-QSAR model and COMSIA method can reflect the relationship between molecular structure and activity more accurately than the traditional 2D-QSAR model, thereby improving the efficiency of drug design. Through fine structural optimization, the derivatives in this study exhibited higher inhibitory activity and selectivity than existing MAO-B inhibitors, helping to reduce side effects and improving the therapeutic efficacy. In addition to internal validation, external validation and molecular docking experiments were conducted to ensure the reliability and practical application potential of the study results.

Based on the statistical results and contour plots of the COMSIA model, the study revealed the key structural factors affecting the activity of the compounds, such as hydrophobicity, hydrogen bond donors, and acceptors. This provided a clear direction for the subsequent design of new compounds by introducing or modifying specific functional groups at specific positions to enhance the activity. By predicting the IC50 values of the newly designed compounds and combining them with molecular docking analysis, the study screened out a number of high-potential candidate compounds, such as compound 31.j. This narrowed down the scope for subsequent experimental validation work and improved the research efficiency.

Based on the designed compound structure, the corresponding synthetic route was developed and the synthesis and purification of the compound were carried out. This step formed the basis for experimental validation and ensured the obtainment of a sufficient number and purity of compound samples. The synthesized new compounds were tested for biological activity, especially for MAO-B inhibitory activity. The predictive accuracy of the QSAR model was verified by comparing the IC50 values of different compounds and the most active compounds were screened.

Further pharmacokinetic and toxicological studies were performed on the screened highly active compounds to assess their absorption, distribution, metabolism, and excretion in the body, as well as potential toxic effects. These studies are important for the clinical application of the compounds. Based on the results of experimental validation, the structures of the compounds were further optimized and iteratively designed. By continuously adjusting the structures of the compounds, we aim to obtain drug candidates with higher activity, lower toxicity, and being more suitable for clinical application.

With respect to the molecular docking limitation aspect, different docking algorithms may use different search strategies and scoring functions, which may lead to differences in docking results [41]. We will analyze currently used docking algorithms, explore their limitations in dealing with complex intermolecular interactions, and consider the introduction or development of more advanced algorithms in the future. Molecules exist in a variety of conformations in solution, and only one or a few low-energy conformations are typically considered in the docking process. We will investigate how the conformational diversity of molecules can be considered more efficiently to improve docking accuracy. Solvent molecules have an important influence on intermolecular interactions in organisms, but current molecular docking methods often ignore this. We will explore how solvent effects can be taken into account during docking to more realistically model the environment of intermolecular interactions. Biological macromolecules, such as proteins, typically undergo conformational changes when binding ligands, while current docking methods tend to treat receptors as rigid structures. We will investigate how to account for receptor flexibility during docking to more accurately predict intermolecular binding modes. Although molecular docking is a powerful tool, its results still need to be confirmed by experimental validation. We will strengthen our experimental validation efforts to assess the reliability and utility of docking results.

In this study, we designed and synthesized a series of novel derivatives based on 6-hydroxybenzothiazole-2-carboxamide, possessing novel chemical structures and offering the possibility of developing novel MAO-B inhibitors. Through Quantitative Structure-activity Relationship (QSAR) modeling and molecular docking studies, we screened a series of highly selective and potent MAO-B inhibitor candidates, which exhibited highly selective inhibition of MAO-B in *in vitro* experiments, and the inhibitory activity was significantly better than that of existing drugs. The novel MAO-B inhibitors developed in this study are expected to overcome the limitations of existing drugs, such as improved efficacy, reduced side effects, and enhanced selectivity, and provide new options for the treatment of neurodegenerative diseases, such as Parkinson's disease and Alzheimer's disease. The effective modeling and prediction basis established in this study may not only provide technical support for the development of novel MAO-B inhibitors, but also accelerate the drug development process and reduce the development cost and time.

## CONCLUSION

The optimal prediction model offered technical assistance for the development of novel structures. A total of two hundred novel derivatives of 6-hydroxybenzothiazole-2-carboxamide were created using compound 31 as a reference. The COMSIA model was used to estimate their IC50 values. Out of all the recently created compounds, compound 31.j exhibited the highest projected IC50 value and had the greatest docking score with the target MAO-B. Therefore, our work offered practical advice for creating new MAO-B inhibitors that can specifically target neurodegenerative lesions to promote neuroprotective effects.

## Figures and Tables

**Fig. (2):**
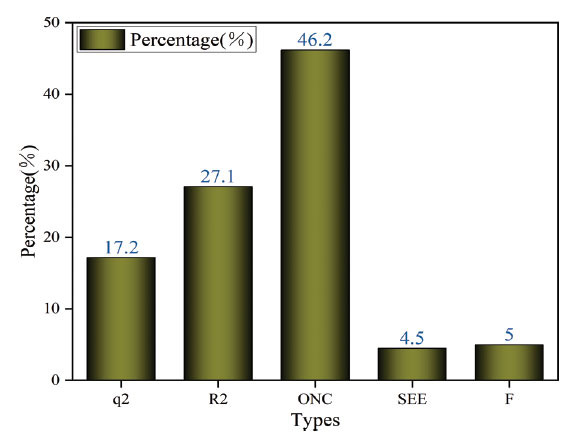
The force field contribution of the most optimal COMSIA model.

**Fig. (3):**
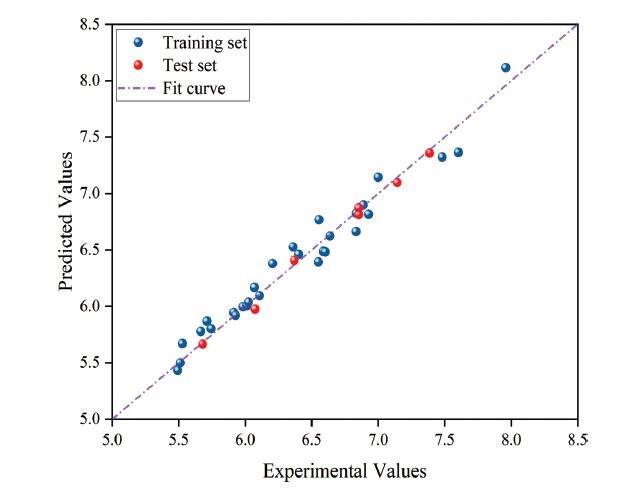
Plot comparing the predicted and experimental values of COMSIA model compounds.

**Fig. (5):**
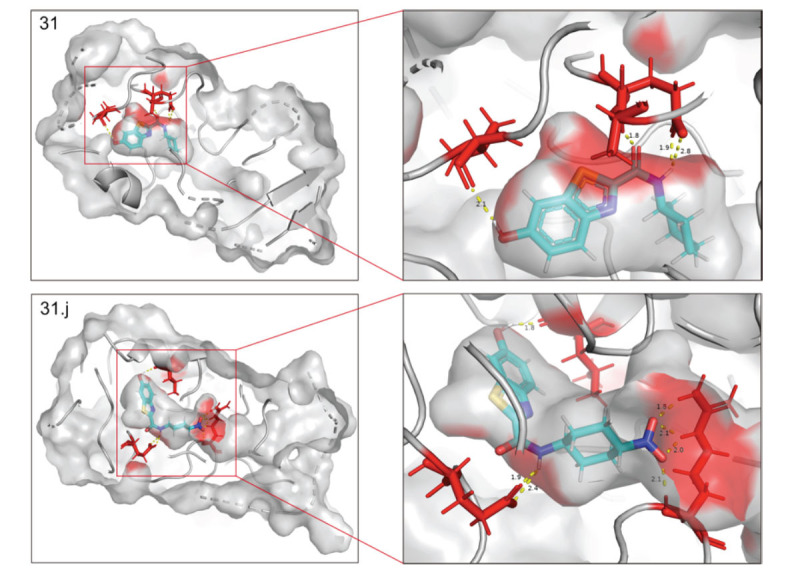
Molecular docking of small molecule compounds 31 and 31.j with MAO-B target molecules being closely related.

**Table 1:** Experimental and projected data for the development of the COMSIA model.

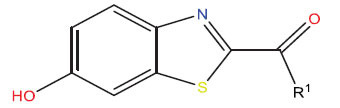					
**Compound**	**R^1^**	**IC50 (μM)**	**Experimental**	**Predicted**	**Residual**
1	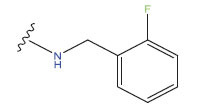	0.279±0.036	6.554	6.865	-0.214
2	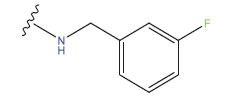	0.622±0.043	6.206	6.651	-0.173
3	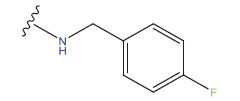	0.397±0.051	6.401	6.484	-0.06
4	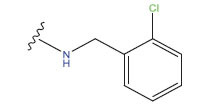	0.146±0.018	6.836	6.788	0.011
5	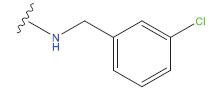	0.437±0.053	6.360	6.682	-0.167
6	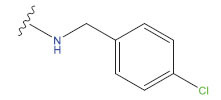	2.97 ± 0.50	5.527	6.342	-0.143
7	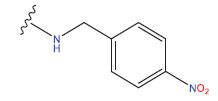	1.04 ± 0.02	5.983	5.982	-0.014
8	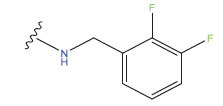	0.025±0.001	7.602	7.249	0.237
9	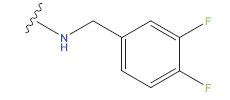	0.033±0.003	7.482	6.856	0.159
10	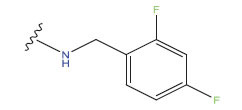	0.100±0.011	7	7.056	-0.145
11	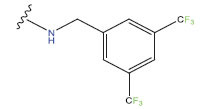	0.129±0.012	6.889	6.838	-0.009
12	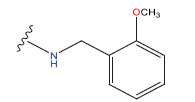	0.258±0.088	6.588	6.376	0.101
13	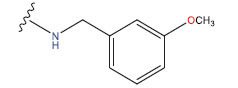	1.94 ± 0.38	5.712	5.791	-0.148
14	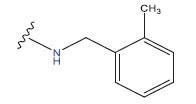	0.147±0.035	6.833	6.373	0.17
15	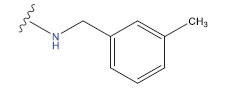	0.282±0.080	6.550	6.177	0.156
16	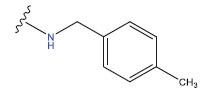	2.16 ± 0.14	5.666	6.03	-0.112
17	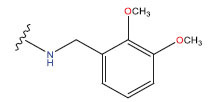	1.22 ± 0.03	5.914	5.89	-0.032
18	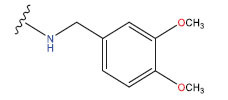	3.21 ± 0.55	5.494	5.33	0.061
19	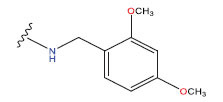	0.971±0.107	6.013	5.966	0.007
20	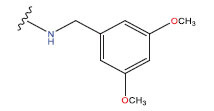	1.80 ± 0.10	5.745	5.675	-0.056
21	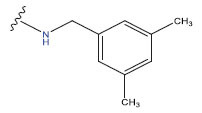	0.230±0.027	6.638	6.293	0.014
22	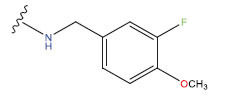	0.851±0.123	6.070	6.163	-0.098
23	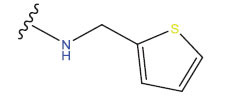	0.250±0.001	6.602	6.364	0.119
24	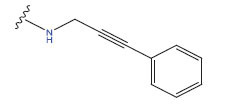	0.944±0.116	6.025	6.14	-0.013
25*	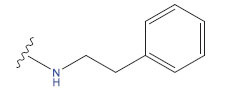	0.041±0.005	6.5544	5.79	0.028
26*	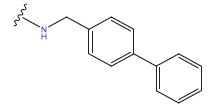	2.09 ± 0.18	6.2062	6.01	0.015
27	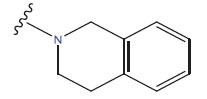	1.18 ± 0.01	7.3872	7.566	0.009
28*	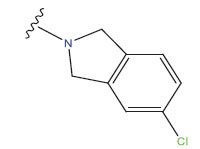	0.140±0.015	6.401	6.624	0.04
29*	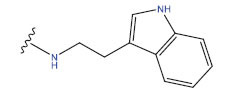	0.140±0.016	6.836	6.571	-0.018
30*	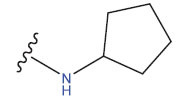	0.842±0.067	6.360	5.796	0.099
31	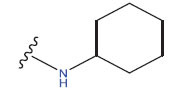	0.011±0.005	5.680	5.783	-0.156
32	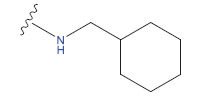	0.118±0.005	5.928	5.998	0.109
33*	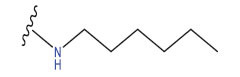	0.072±0.008	5.983	6.18	0.046
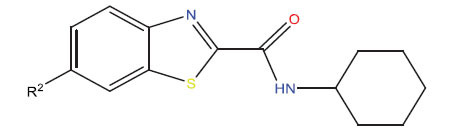					
**Compound**	**R^2^**	**IC50 (μM)**	**Experimental**	**Predicted**	**Residual**
34*	-OCH_3_	0.428±0.019	7.602	6.862	-0.041
35	-H	3.08 ± 0.25	6.854	6.895	0.013
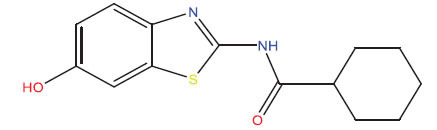					
**Compound**	**-**	**IC50 (μM)**	**Experimental**	**Predicted**	**Residual**
36	-	0.779±0.018	6.854	6.791	0.013

**Note: ** * the test set data.

**Table 2:** Statistical parameters of the COMSIA model.

**No.**	**Model**	**q^2^**	**ONC**	**r^2^**	**F-value**	**SEE**	**r^2^_pre_**
1	ESHAD	0.569	3	0.915	52.714	0.109	5.783
2	ESA	0.537	4	0.861	53.130	0.167	5.736
3	EHD	0.369	5	0.921	44.644	0.101	5.737
4	ESD	0.466	4	0.856	52.597	0.172	5.605
5	ESHA	0.551	3	0.915	52.720	0.109	5.654
6	ESHD	0.528	3	0.913	52.129	0.112	5.681
7	EHAD	0.339	4	0.922	54.834	0.101	5.729
8	SHAD	0.552	4	0.780	37.428	0.236	5.722
9	ESAD	0.499	5	0.857	42.624	0.171	5.601
10	ESH	0.516	4	0.912	51.785	0.113	5.658

**Note: ** S: steric, E: electrostatic, H: hydrophobic, D: hydrogen bond donor, A: hydrogen bond acceptor.

**Table 3:** The recently developed compounds together with their projected values.

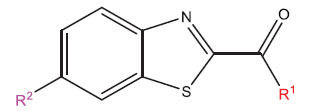				
**No.**	**R^1^**	**R^2^**	**pIC50 Predicted by COMSIA**	**Total Scores**
31	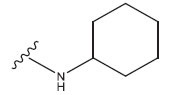	-OH	5.783	6.0648
31.a	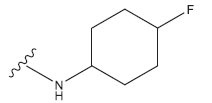	-OH	6.431	5.3026
31.b	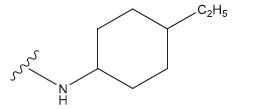	-OH	6.386	6.0734
31.c	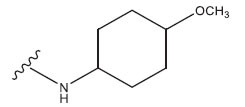	-OH	6.375	6.3952
31.d	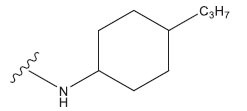	-OH	6.359	7.2428
31.e	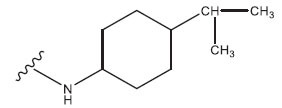	-OH	6.452	7.8233
31.f	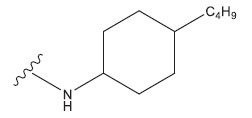	-OH	6.355	6.2652
31.g	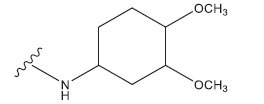	-OH	6.247	6.4829
31.h	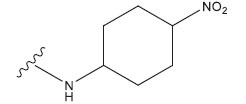	-OCH3	5.739	6.9519
31.i	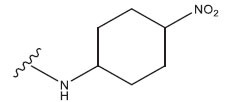	-H	6.464	6.4516
31.j	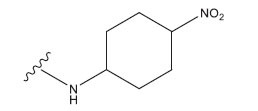	-OH	6.391	8.5509

## Data Availability

The data and supportive information are available within the article.

## LIST OF ABBREVIATIONS

COMSIA: Comparative molecular similarity index analysis
QSAR: Quantitative structure-activity relationship
